# The antibacterial properties of Malaysian tualang honey against wound and enteric microorganisms in comparison to manuka honey

**DOI:** 10.1186/1472-6882-9-34

**Published:** 2009-09-15

**Authors:** Hern Tze Tan, Rosliza Abdul Rahman, Siew Hua Gan, Ahmad Sukari Halim, Siti Asma' Hassan, Siti Amrah Sulaiman, Kirnpal-Kaur BS

**Affiliations:** 1 Department of Medical Microbiology and Parasitology, School of Medical Sciences, Health Campus, Universiti Sains Malaysia, Kubang Kerian 16150, Kelantan, Malaysia; 2 Department of Pharmacology, School of Medical Sciences, Health Campus, Universiti Sains Malaysia, Kubang Kerian 16150, Kelantan, Malaysia; 3 Reconstructive Sciences Unit, School of Medical Sciences, Health Campus, Universiti Sains Malaysia, Kubang Kerian 16150, Kelantan, Malaysia

## Abstract

**Background:**

Antibiotic resistance of bacteria is on the rise, thus the discovery of alternative therapeutic agents is urgently needed. Honey possesses therapeutic potential, including wound healing properties and antimicrobial activity. Although the antimicrobial activity of honey has been effectively established against an extensive spectrum of microorganisms, it differs depending on the type of honey. To date, no extensive studies of the antibacterial properties of tualang (*Koompassia excelsa*) honey on wound and enteric microorganisms have been conducted. The objectives of this study were to conduct such studies and to compare the antibacterial activity of tualang honey with that of manuka honey.

**Methods:**

Using a broth dilution method, the antibacterial activity of tualang honey against 13 wound and enteric microorganisms was determined; manuka honey was used as the control. Different concentrations of honey [6.25-25% (w/v)] were tested against each type of microorganism. Briefly, two-fold dilutions of honey solutions were tested to determine the minimum inhibitory concentration (MIC) against each type of microorganism, followed by more assays within a narrower dilution range to obtain more precise MIC values. MICs were determined by both visual inspection and spectrophotometric assay at 620 nm. Minimum bactericidal concentration (MBC) also was determined by culturing on blood agar plates.

**Results:**

By visual inspection, the MICs of tualang honey ranged from 8.75% to 25% compared to manuka honey (8.75-20%). Spectrophotometric readings of at least 95% inhibition yielded MIC values ranging between 10% and 25% for both types of honey. The lowest MBC for tualang honey was 20%, whereas that for manuka honey was 11.25% for the microorganisms tested. The lowest MIC value (8.75%) for both types of honey was against *Stenotrophomonas maltophilia*. Tualang honey had a lower MIC (11.25%) against *Acinetobacter baumannii *compared to manuka honey (12.5%).

**Conclusion:**

Tualang honey exhibited variable activities against different microorganisms, but they were within the same range as those for manuka honey. This result suggests that tualang honey could potentially be used as an alternative therapeutic agent against certain microorganisms, particularly *A. baumannii *and *S. maltophilia*.

## Background

Since ancient times, honey has been used for its medicinal properties to treat a wide variety of ailments. In particular, it has been used in wound dressings. In general, all types of honey have high sugar content but a low water content and acidity, which prevent microbial growth. Most types of honey generate hydrogen peroxide when diluted because of the activation of the enzyme glucose oxidase, which oxidizes glucose to gluconic acid and hydrogen peroxide [1,2]. Hydrogen peroxide is the major contributor to the antimicrobial activity of honey, and the different concentrations of this compound in different honeys result in their varying antimicrobial effects [1,3].

In most cases, the peroxide activity in honey can be destroyed easily by heat or the presence of catalase. However, *Leptospermum *honeys retain their antimicrobial activities even in the presence of catalase, thus they are known as "non-peroxide honeys" [3]. Several components may contribute to the non-peroxide activities, such as the presence of methyl syringate and methylglyoxal, which have been extensively studied in *Leptospermum *honeys [4-6]. However, many other constituents that have yet to be characterized are likely to contribute to honey's antimicrobial properties.

Honey can inhibit the growth of a wide range of bacteria, fungi, protozoa and viruses [3,7]. Microorganisms such as *Staphylococcus aureus*, *Pseudomonas aeruginosa *and *Escherichia coli *frequently are isolated from skin wounds. Methicillin-resistant *S. aureus *(MRSA) is involved in difficult-to-treat skin and underlying tissue infections associated with Gram-positive bacteria [8], while the most serious complication in burn patients is associated with infection with *P. aeruginosa *[9,10], followed by infections with *E. coli*, *S. aureus *and other pathogenic microorganisms [9]. Microorganisms that colonize a burn wound originate from the patient's endogenous skin, gastrointestinal and respiratory flora and via contact with contaminated external environmental surfaces, water, air and the soiled hands of health care workers. Gram-positive bacteria from the patient's endogenous skin flora or the external environment predominantly colonize the burn wound immediately, followed by endogenous Gram-negative bacteria from the patient's gastrointestinal flora in the first few days after injury [11].

Besides its antimicrobial properties, honey can clear infection in a number of ways, including boosting the immune system, having anti-inflammatory and antioxidant activities and via stimulation of cell growth [12]. The vast amount of data about honey's therapeutic properties, along with the rapidly increasing interest in and research into natural health remedies and supplements, has led to a resurgence in interest in honey's therapeutic uses.

Malaysian tualang honey is collected from the combs of Asian rock bees (*Apis dorsata*), which build their hives high up in the tualang tree (*Koompassia excelsa*). Tualang honey is used commonly as a medicinal product [13,14] and as food in Malaysia. However, little scientific information about its microbiological properties has been published to date. Thus, this study was designed to determine the antibacterial activity of tualang honey by comparing it with manuka honey, which has been extensively studied [3,5].

Previously, Ainul Hafiza *et al. *[13] conducted a study of five local honeys (Belimbing, Gelam, Durian, Kelapa and Tualang); they conducted a microbial colony count using a filtration method and a simple screening assay for antibacterial action against *Staphylococcus aureus *using the agar diffusion method. Tumin *et al. *[15] investigated the antibacterial properties of tualang honey and four other local Malaysian honeys against six bacterial species. In our study, we examined the antibacterial activity of the local Malaysian tualang honey against 13 different bacterial species and compared it with the activity of manuka honey. To our knowledge, this is the first study to reveal the broad spectrum of antibacterial activities of the local Malaysian tualang honey.

## Methods

The antibacterial properties of tualang honey against 13 different bacterial species were determined by comparison to the commercially available manuka honey (Kordel's, UMF10+). Malaysian tualang honey was obtained from Federal Agriculture Marketing Authority (FAMA), Kedah, Malaysia, which was collected from *Koompassia excelsa *(tualang tree). Both types of honey were stored in the dark at room temperature. Initially, the honeys were subjected to sterilization by γ-irradiation at a dose of 25 kGy followed by a sterility test before they were subjected to the various antimicrobial tests. We used this radiation dose because it has been shown not to cause significant loss of antibacterial activity [16] and because no viable clostridial spores are present after the process [16,17].

The minimum inhibitory concentration (MIC) of the honey was determined using the broth dilution method in sterile 48-well microtiter plates with lids (Nunc, Roskilde, Denmark). Fifty percent (w/v) stock solution of each type of honey was prepared by weighing 10 g of the honey and bringing the volume up to 20 ml using cation-adjusted Mueller Hinton II broth (CAMHB) (Becton Dickinson, Maryland, USA). Further dilutions were done to obtain honey concentrations of 6.25% (w/v), 7.5%, 8.75%, 10%, 11.25%, 12.5%, 15%, 17.5%, 20%, 22.5% and 25%. The lowest concentration of honey that prevented the growth of each microorganism, as detected by lack of visual turbidity compared to a negative control, was recorded as the MIC. All honey solutions were freshly prepared before each assay.

The following procedure was followed for each microorganism with each type of honey. A few single bacterial colonies from an overnight culture on blood agar (BA) were inoculated into peptone water to achieve a turbidity of 0.5 McFarland (≈ 1 × 10^8 ^CFU/ml). The bacterial suspension was further diluted with CAMHB to obtain a final concentration of inoculum of 5 × 10^5 ^CFU/ml. The final volume in each test well was 1 ml, consisting of 0.5 ml diluted honey and 0.5 ml bacterial inoculum. For each assay, control wells included: 1) wells containing broth only (without honey and inoculum); 2) wells containing broth and inoculum (without honey); and 3) wells containing broth and honey (without inoculum). The microtiter plates were incubated at 35°C for 18 h. All tests were performed in triplicate and were repeated three times to obtain reliable results.

Growth was observed by visual inspection and by measuring the optical density (OD) at 620 nm using a spectrophotometer (VERSAmax, Massachusetts, USA). The OD was measured immediately after the visual reading. The growth inhibition for the test wells at each honey dilution was determined by the formula: Percent inhibition = [1 - (OD test well - OD corresponding negative control well)/(OD viability control well - OD broth only well)] × 100%. The minimum and maximum values were 0% and 100%, respectively.

The microorganisms tested included five Gram-positive bacteria [*Streptococcus pyogenes *(ATCC 19615), coagulase-negative Staphylococci [local clinical isolates (l.c.i.)], Methicillin-resistant *Staphylococcus aureus *(MRSA) (ATCC 33591), *Streptococcus agalactiae *(l.c.i.) and *Staphylococcus aureus *(ATCC 25923)] and eight Gram-negative bacteria [*Stenotrophomonas maltophilia *(l.c.i.), *Acinetobacter baumannii *(l.c.i.), *Salmonella enterica *Serovar *typhi *(l.c.i.), *Pseudomonas aeruginosa *(ATCC 27853), *Proteus mirabilis *(l.c.i.), *Shigella flexneri *(l.c.i.), *Escherichia coli *(ATCC 25922) and *Enterobacter cloacae *(l.c.i.)].

The minimum bactericidal concentration (MBC) was determined by taking a loopful of the culture medium from each test well (from the broth MIC assay) that showed no apparent growth and sub-culturing on fresh BA plates. After incubation at 35°C for 24 h, the MBC was read as the least concentration showing no growth on the BA plates.

Non-peroxide activity of both tualang and manuka honeys were screened using the agar well diffusion method adapted from Allen *et al*. [18] against *S. aureus *cultured on the surface of Mueller Hinton agar. The 50% (w/v) solutions of each honey were diluted to 25% (w/v) by taking 1 ml of each and adding it to either 1 ml of sterile purified water (total activity) or 1 ml of catalase solution (non-peroxide activity). An 8000 U catalase solution (Sigma, C9322: 2950 units/mg) was used to remove all the hydrogen peroxide present in the honeys. The removal of hydrogen peroxide was verified according to the method described [18]. Blanks of water and catalase solution were included.

## Results

In this study, we compared the MIC values of tualang honey and manuka honey determined by visual inspection and by a spectrophotometer. The MBC values of tualang and manuka honeys also were compared.

Under visual inspection, the MICs for tualang honey ranged from 8.75% (w/v) to 25%, while those for manuka honey ranged between 8.75% and 20% (Table 1). Manuka honey had lower MICs (indicating better activity) than tualang honey against nine of the tested bacteria (*S. pyogenes*, coagulase-negative Staphylococci, MRSA, *S. agalactiae*, *S. aureus*, *P. mirabilis*, *S. flexneri*, *E. coli *and *E. cloacae*). However, when tested against *A. baumannii*, tualang honey had a better MIC value (11.25%) compared to that of manuka honey (12.5%). When tested against *S. typhi *and *P. aeruginosa*, both honey types had equal MIC values (15% and 17.5%, respectively). Both honey types had the lowest MIC value (8.75%) and thus the best activity, against *S. maltophilia*. Additionally, the 8.75% dilution of manuka honey showed good inhibition against the growth of MRSA. The highest MIC values for tualang and manuka honeys were against *E. cloacae*, at 25% and 20% respectively. Tualang (20%) and manuka (11.25%) honeys exhibited the greatest difference in MIC value against *S. aureus*.

**Table 1:** MIC values (%) determined by visual inspection and spectrophotometric measurement

**No.**	**Microorganism**	**Visual MIC (%)**		**Spectrophotometric MIC_95 _(%)**	
			
		**Tualang**	**Manuka**	**Tualang**	**Manuka**
1	*Streptococcus pyogenes*	12.5	11.25	12.5	12.5
2	Coagulase-negative Staphylococci	12.5	11.25	11.25	12.5
3	Methicillin-resistant *Staphylococcus aureus*	12.5	8.75	11.25	12.5
4	*Streptococcus agalactiae*	20	15	20	15
5	*Staphylococcus aureus*	20	11.25	17.5	12.5
					
6	*Stenotrophomonas maltophilia*	8.75	8.75	10	10
7	*Acinetobacter baumannii*	11.25	12.5	10	12.5
8	*Salmonella enterica *Serovar *typhi*	15	15	15	17.5
9	*Pseudomonas aeruginosa*	17.5	17.5	17.5	17.5
10	*Proteus mirabilis*	20	17.5	22.5	22.5
11	*Shigella flexneri*	20	17.5	25	25
12	*Escherichia coli*	22.5	17.5	22.5	20
13	*Enterobacter cloacae*	25	20	25	22.5

Spectrophotometric readings of at least 95% inhibition (MIC_95_) gave MIC values that ranged between 10% and 25% for both honey types. Tualang honey had lower MIC_95 _values than manuka honey when tested against coagulase-negative Staphylococci, MRSA, *A. baumannii *and *S. typhi*. However, manuka honey had lower MIC_95 _values compared to tualang honey when tested against *S. agalactiae, S. aureus, E. coli *and *E. cloacae*. For the remaining bacterial species, both types of honeys demonstrated similar antimicrobial activities (Table 1).

For tualang honey, MIC_95 _values calculated from spectrophotometric readings were the same as MIC values determined by visual inspection for six bacteria (*S. pyogenes, S. agalactiae, S. typhi, P. aeruginosa, E. coli *and *E. cloacae*). However, four bacteria (coagulase-negative Staphylococci, MRSA, *S. aureus *and *A. baumannii*) had slightly lower (1.25-2.5%) spectrophotometric MIC_95 _values than visually determined MIC values, whereas three bacteria (*S. maltophilia, P. mirabilis *and *S. flexneri*) had increased (1.25-5%) spectrophotometric MIC_95 _values compared to visual MIC values.

For manuka honey, spectrophotometric MIC_95 _and visual MIC values were the same for three bacteria (*S. agalactiae*, *A. baumannii *and *P. aeruginosa*). The other ten bacteria tested had greater spectrophotometric MIC_95 _(1.25-7.5%) values compared to visual MIC values; the biggest difference between the two methods (7.5%) was seen with *S. flexneri*.

Manuka honey had lower MBC values compared to tualang honey (Table 2). Nine of the bacteria tested required a concentration of greater than 25% tualang honey to kill them, whereas only four species of bacteria needed this concentration of manuka honey to be killed. The lowest MBC value for tualang honey was 20% (against *S. typhi*), whereas for manuka honey it was 11.25% (against *S. maltophilia*).

**Table 2:** MBC values (%) of tualang honey and manuka honey

**No.**	**Microorganism**	**MBC (%)**	
		
		**Tualang**	**Manuka**
1	*Streptococcus pyogenes*	25	25
2	Coagulase-negative Staphylococci	>25	>25
3	Methicillin-resistant *Staphylococcus aureus*	>25	25
4	*Streptococcus agalactiae*	>25	22.5
5	*Staphylococcus aureus*	>25	25
			
6	*Stenotrophomonas maltophilia*	25	11.25
7	*Acinetobacter baumannii*	>25	12.5
8	*Salmonella enterica *Serovar *typhi*	20	17.5
9	*Pseudomonas aeruginosa*	25	22.5
10	*Proteus mirabilis*	>25	>25
11	*Shigella flexneri*	>25	>25
12	*Escherichia coli*	>25	17.5
13	*Enterobacter cloacae*	>25	>25

**Figure 1:**
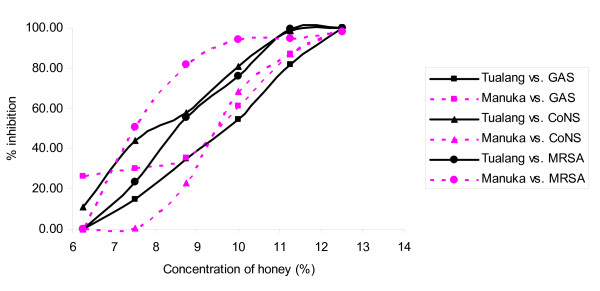
**Inhibition of the growth of *S. pyogenes *(GAS), coagulase-negative Staphylococci (CoNS) and MRSA**.

**Figure 2:**
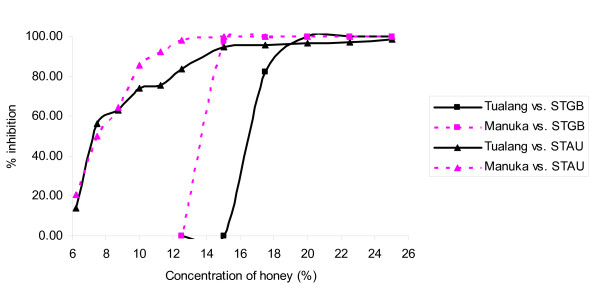
**Inhibition of the growth of *S. agalactiae *(STGB) and *S. aureus *(STAU)**.

**Figure 3:**
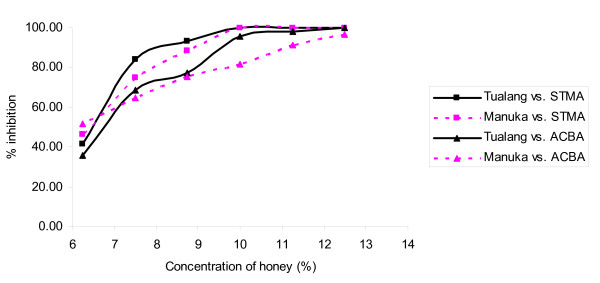
**Inhibition of the growth of *S. maltophilia *(STMA) and *A. baumannii *(ACBA) caused by tualang and manuka honeys at different concentrations**.

**Figure 4:**
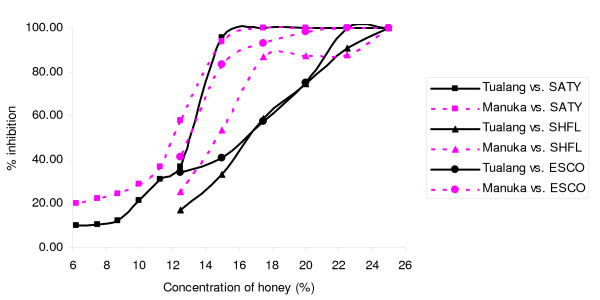
**Inhibition of the growth of *S. typhi *(SATY), *S. flexneri *(SHFL) and *E. coli *(ESCO)**.

**Figure 5:**
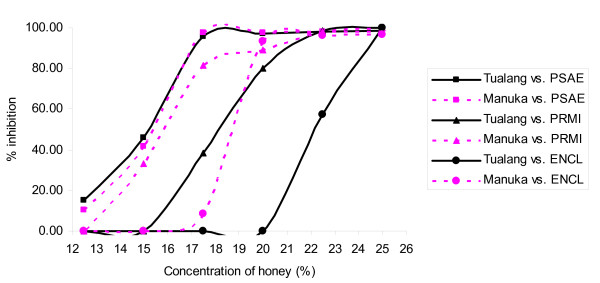
**Inhibition of the growth of *P. aeruginosa *(PSAE), *P. mirabilis *(PRMI) and *E. cloacae *(ENCL)**.

Figures 1, 2, 3, 4 &5 show the patterns of bacterial growth inhibition caused by exposure to different concentrations of tualang and manuka honeys. Figure 1 shows that both honeys caused similar inhibition of *S. pyogenes *growth but tualang honey inhibited growth of coagulase-negative Staphylococci better than manuka. Manuka honey caused greater inhibition of MRSA growth than tualang honey at concentrations from 6.25% to 11%, after which tualang honey was the better inhibitor. Figure 2 illustrates that both honeys caused similar inhibition of *S. agalactiae *and *S. aureus *growth. The inhibition of *S. aureus *caused by tualang honey was more gradual compared to that of manuka honey.

Figure 3 shows similar patterns of inhibition of *S. maltophilia *by both honeys. For *A. baumanni*, tualang honey caused greater inhibition of growth from 7.5% onwards compared to manuka honey. Both honeys caused similar patterns of inhibition of *S. typhi *growth, with a gradual increase followed by a sharp increase in inhibition around 11.25% to 15% (Figure 4). Manuka honey inhibited *S. flexneri *growth better than tualang honey from 12.5% to 21.5%, but at greater concentrations tualang honey was the better inhibitor. The effect of the honeys was different for *E. coli*. Figure 5 shows that both honeys caused similar patterns of growth inhibition of *P. aeruginosa*. For *P. mirabilis*, manuka honey was a better inhibitor than tualang honey from 12.5% to 22.5%. Inhibition of *E. cloacae *growth began at 16.5% manuka honey and 20% tualang honey, followed by drastic increase of inhibition.

The agar well diffusion assays performed showed that the total activity and non-peroxide activity of the honeys were similar, in which the inhibition zone diameter measured was 24 mm for tualang honey and 26 mm for manuka honey. However, it was noted that there was a thin layer of growth of the organism in the zone of inhibition.

## Discussion

Tualang honey is readily available in Malaysia, but its quality and floral origin have yet to be determined and standardized. In contrast, manuka honey has been widely researched and its antibacterial potential is renowned worldwide. Honeys with proven antibacterial potency (UMF 10+ and above) have been recommended for wound care preferentially over honeys of low or unknown potency [19]. Therefore, manuka honey with UMF 10+ was chosen as a comparison for this study of the antimicrobial activity of tualang honey.

In this study, we found that tualang honey has variable but broad-spectrum activities against many different species of wound and enteric bacteria. Its activity was comparable to that of manuka honey when tested against certain bacterial species. Lusby *et al*. [20] reported that honeys other than the commercially available antibacterial honeys (e.g., manuka honey) can have equivalent antibacterial activity against some bacteria, whereas Basson and Grobler [21] found no exceptionally high antimicrobial activity of honeys from indigenous wild flowers from South Africa.

We chose the broth dilution method for this study because it generates more quantitative and precise results compared to the agar well diffusion method. Moreover, the MIC values determined by the broth dilution method were lower (indicating higher activity) than those obtained using the agar well diffusion method, as diffusion rates of active constituents in agar may be slower than those in broth [22].

We also performed spectrophotometric assays using microtiter plates; it is a simple and rapid method, it has a greater sensitivity than the standard well and disc diffusion methods and the results are highly reproducible [23]. Spectrophotometry can detect inhibitory levels below those recorded for well or disc diffusion assays [23]. In our study, visual inspection might not have been accurate because impurities in the honeys (especially in tualang honey) might have caused disturbance and imprecision in the readings. Moreover, a medium containing bacterial growth not detectable by eye would have been described as clear by visual inspection, but the growth would have been detectable spectrophotometrically. These might have caused the wide variation seen in MIC values determined by visual inspection and by spectrophotometric measurement (e.g., our results for *S. flexneri*). Visual inspection also could not distinguish the percentage of growth in the turbid wells. MIC determination by visual inspection might also vary from person to person depending on the eye of the observer.

Having known that the presence of hydrogen peroxide in honeys contributes to its antibacterial activity, we screened the non-peroxide activity of both tualang and manuka honeys and found that the antibacterial activity persisted after the addition of catalase for both honeys, suggesting the presence of non-peroxide activity.

Analysis of the inhibition of bacterial growth caused by different honey concentrations revealed both differences and similarities in the pattern of inhibition exhibited by the 13 microorganisms tested in this study. Most bacteria showed similar growth inhibition patterns for both honeys tested, but some variations were detected. The observed differences might reflect how each type of bacteria reacts to honey treatment.

Tualang and manuka honeys showed good antimicrobial activity against *S. maltophilia*; both honeys yielded the lowest visual MIC of 8.75% (w/v) against this microorganism. This organism is an aerobic, non-fermentative, Gram-negative bacterium that causes uncommon but difficult to treat infections in immunocompetent individuals, such as pneumonia, urinary tract infection and blood stream infection. It can lead to nosocomial infections and latent pulmonary infections in immunocompromised patients [24]. *S. maltophilia *often is difficult to eradicate because it is naturally resistant to many broad-spectrum antibiotics, including all carbapenems; moreover, increasing resistance has been reported for co-trimoxazole and ticarcillin [25]. Thus, the use of tualang honey to treat *S. maltophilia *infections should be further investigated.

When compared to manuka honey, tualang honey exhibited better antimicrobial activity against *A. baumannii*. *A. baumannii *is a pathogenic, aerobic, Gram-negative bacillus and most of its isolates are inherently multi-drug resistant [26]. *A. baumannii *is an opportunistic pathogen that usually infects immunocompromised individuals through open wounds, catheters and breathing tubes. Multi-drug resistant *A. baumannii *has emerged as a major cause of nosocomial infection, with resistance against its first line of treatment (i.e., carbapenems such as imipenem) [27]. Therefore, sterilized tualang honey has the potential to be used as an alternative agent for wound infection caused by this bacterium. However, this approach needs to be further studied.

Haffejee and Moosa [28] discovered that honey is effective in treating bacterial gastroenteritis in infants. Honey was reported to be effective when used as a substitute for glucose in oral rehydration and its antibacterial activity shortened the duration of bacterial diarrhoea. In our study, the growth of bacterial species that cause gastric infections, such as *S. typhi*, *S. flexneri *and *E. coli*, were inhibited by tualang honey at concentrations between 15% and 22.5% (w/v). Previous preliminary study on tualang honey also reported that it has antibacterial activity against *E. coli, S. typhi *and *S. pyogenes *[15]. Thus, when taken orally in its pure undiluted form, tualang honey may help speed up recovery from such infections.

The spectrophotometric data obtained in this study revealed that tualang honey had slightly better inhibitory activity than manuka honey against MRSA. This activity may turn out to be quite beneficial, as there has been a discernible increase in difficult-to-treat skin and underlying tissue infections associated with Gram-positive bacteria like MRSA. Thus, more effective treatment is needed to treat MRSA [8].

The MBC values obtained in this study indicated the minimum concentration of honey needed to kill 99.9% of bacteria. However, the indications for determination of bactericidal activity are rare and are usually meant for serious infections, such as in immunocompromised patients or infections at a site that is difficult to be reached with available antibiotics. In this study, the MBC of tualang honey was remarkable against one Gram-positive bacterium (*S. pyogenes*) and three Gram-negative bacteria (*S. maltophilia, S. typhi *and *P. aeruginosa*).

Most deaths in severely burn-injured patients are due to burn wound sepsis or complications due to inhalation injury. Currently, the emerging antimicrobial resistance trends in burn wound bacterial pathogens are a serious challenge [11]. Thus, honey with effective antimicrobial properties against antibiotic-resistant organisms such as MRSA and multiple-resistant Gram-negative rods such as *Pseudomonas aeruginosa*, *Acinetobacter *spp. and members of the family *Enterobacteriaceae*, which have been associated with infections of burn wounds and sites of major thermal injury and in nosocomial infections, is much anticipated [11,29].

## Conclusion

Tualang honey exhibited variable activities against many different microorganisms. In some cases it showed equivalent or better activities than manuka honey, especially against *S. maltophilia *and *A. baumannii*. The potency of tualang honey against certain microorganisms suggests its potential to be used as an alternative therapeutic agent for certain medical conditions, particularly wound infection.

## Competing interests

The authors declare that they have no competing interests.

## Authors' contributions

HTT carried out the antibacterial assays, performed the analysis of data, interpretation of results and drafted the manuscript. RAR participated in the collection, identification and maintenance of the pure bacterial strains. SHG participated in the design of the study and edited the manuscript. ASH participated in obtaining the bacterial strains from wound infections and burn patients and edited the draft. SAH and SAS were involved in the acquisition of the grant and design of the study. KKBS participated in the acquisition of funding, design of the study, coordination and monitoring of research and edited the manuscript. All authors have read and approved the final manuscript.

## Pre-publication history

The pre-publication history for this paper can be accessed here:


